# Effects of Selenium and Cadmium on Breast Muscle Fatty-Acid Composition and Gene Expression of Liver Antioxidant Proteins in Broilers

**DOI:** 10.3390/antiox8050147

**Published:** 2019-05-27

**Authors:** Evangelos Zoidis, George Papadomichelakis, Athanasios C. Pappas, Georgios Theodorou, Kostas Fegeros

**Affiliations:** 1 Department of Nutritional Physiology and Feeding, Faculty of Animal Science, Agricultural University of Athens, 11855 Athens, Greece; gpapad@aua.gr (G.P.); apappas@aua.gr (A.C.P.); cfeg@aua.gr (K.F.); 2 Department of Animal Breeding and Husbandry, Faculty of Animal Science, Agricultural University of Athens, 11855 Athens, Greece

**Keywords:** antioxidant enzymes, cadmium, fatty acids, gene expression, selenium, selenoproteins

## Abstract

The present work was part of a project intended to evaluate whether organic selenium (Se) has the potential to protect against toxic effects exerted by cadmium (Cd). For this reason, 300 as-hatched, one-day-old broiler chickens were randomly allocated in four dietary treatments with five replicate pens per treatment. Chickens in T1 treatment, were offered a diet supplemented with 0.3 ppm Se (as Se-yeast), without added Cd; in T2 treatment, they were offered a diet with 0.3 ppm Se and 10 ppm Cd; in T3 treatment, they were offered a diet with 0.3 ppm Se and 100 ppm Cd; in T4 treatment, chickens were offered a diet supplemented with 3 ppm Se and 100 ppm Cd. Cadmium was added to the diets in T2, T3, and T4 as CdCl_2_. On the fourth and sixth weeks, liver and breast samples were obtained from two broilers per replicate pen. Relative gene expression levels of catalase (CAT), superoxide dismutase 1 (SOD1) and 2 (SOD2), methionine sulfoxide reductase A (MSRA) and B3 (MSRB3), iodothyronine deiodinase 1 (DIO1), 2 (DIO2), and 3 (DIO3), glutathione peroxidase 1 (GPX1) and 4 (GPX4), thioredoxin reductase 1 (TXNRD1) and 3 (TXNRD3), and metallothionein 3 (MT3) were analyzed by real-time quantitative PCR in liver, whereas the fatty-acid (FA) profile of breast muscle was determined by gas chromatography. Broilers supplemented with 0.3 ppm Se could tolerate low levels of Cd present in the diets, as there were no significant changes in the breast muscle FA profile, whereas excess Cd led to decreased polyunsaturated fatty acids (PUFAs), and in particular *n*-6 PUFA. Furthermore, treatments mainly affected the messenger RNA (mRNA) expression of SOD2, TXNRD3, and MT3, while age affected CAT, MSRB3, DIO2, DIO3, GPX4, TXNRD1, and MT3. In conclusion, dietary Se may help against the negative effects of Cd, but cannot be effective when Cd is present at excessive amounts in the diet.

## 1. Introduction

Cadmium (Cd) is a non-essential heavy metal and one of the most toxic environmental pollutants. Its occurrence in feed constitutes a considerable issue in agriculture and animal production. In some circumstances, Cd levels surpass the maximum permitted limits. There are mainly two routes of animal exposure to Cd including the application of feed mineral premixes with high-Cd residues, and the use of animal manure as an organic fertilizer that contains high levels of Cd [1]. Cadmium is known for its comprehensive toxicity to mammals, and numerous experiments demonstrated that Cd causes various forms of oxidative damage and lesions to animal tissues [2]. It was demonstrated that Cd induced free-radical production, reduced the activities of antioxidant enzymes [3,4], and resulted in oxidative deterioration of lipids [4], proteins, and DNA in humans and animals [2,5,6].

Cadmium-induced hepatotoxicity, oxidative stress, and apoptosis were identified in chicken liver [7], and testicular toxicity was induced by dietary Cd in cocks [8]. Thus, it is believed that antioxidants may serve as useful inhibitors against Cd toxicity. Among antioxidative micronutrients, selenium (Se) is an essential dietary trace element which plays a pivotal role in the protection of toxicity exerted by each of the non-essential heavy metals (As, Pb, Hg, and Cd) [9].

Selenium was shown to play a crucial role in the antioxidant defense system, protecting in this way the organism from oxidative stress [10,11]. Moreover, Se was shown to be an integral component of various enzymes such as glutathione peroxidases, deiodinases, thioredoxin reductases, and other selenoproteins [12]. It was demonstrated that Se plays ameliorative roles against the toxicity of Cd in a variety of animal tissues [6]. Selenium ameliorated the oxidative stress induced by Cd or amended the antioxidant defense system by regulating the expressions of some selenoproteins [13,14]. Therefore, selenoproteins, among other antioxidant enzymes, may play crucial roles in the process of Cd toxicity and the antagonistic actions of Se.

The larger part of research investigating means to reduce Cd toxicity via Se supply was conducted in mammals using inorganic forms of Se [13,15]. Inorganic Se orally provided to suckling rats, in equimolar amounts to Cd, reduced liver and kidney Cd levels of the pups [16]. Furthermore, organic Se is a highly available form of Se for livestock and grants greater levels of antioxidant protection than its inorganic form [17,18].

Various studies showed the ability of Se to protect from Cd toxicity in mammals. In detail, Jihen et al. [15] demonstrated that Se moderately alleviated the damages resulting from Cd toxicity in rat liver and kidney. Simultaneous treatment with Se decreased Cd-induced liver histopathological changes, oxidative stress, and apoptosis. These results proposed that the toxic effects of Cd on the liver were partially alleviated by Se [2]. Selenium supplementation was also shown to modify the distribution of Cd in the liver [14,19]. Chen et al. [20] studied the protective effect of Se on Cd-induced changes of heat-shock protein (HSP) genes. Zhao et al. [6] studied the protective effect of Se on Cd-induced changes of selenoprotein K, N, S, and T genes. Liu et al. [21] reported that Se ameliorated Cd-induced brain damage in chickens by regulating inducible nitric oxide synthase (iNOS)/nitric oxide (NO) system changes. Therefore, Se administration could serve as a potential therapy for Cd-induced negative effects in chickens [21].

The present study was part of a bigger project designed to assess whether organic Se administered at high doses could protect broilers from Cd toxicity. Our previous studies revealed that Se can ameliorate the negative effects of Cd toxicity primarily due of its antioxidant function, and that it can protect against Cd accumulation in the tissues [1,19]. Moreover, supranutritional levels of supplemented Se in chickens, at levels well below those causing toxicity, prevented peroxidation of long-chain polyunsaturated fatty acids (PUFAs), such as eicosapentaenoic acid (EPA) and docosahexaenoic acid (DHA), which are known for their health beneficial effects [22].

However, the effects of Cd/Se supplementation in chicken on breast fatty-acid (FA) contents and hepatic messenger RNA (mRNA) expression of antioxidant enzymes are not reported. The study on gene expression of these antioxidant enzymes in Cd toxicity may provide a useful means to investigate the pathogenesis of Cd toxicity and further elucidate the role of organic Se in the process of alleviation of Cd toxicity.

The aim of the present study was to examine transcriptional changes of the antioxidant proteins and selenoproteins catalase (CAT), superoxide dismutase 1 (SOD1) and 2 (SOD2), methionine sulfoxide reductase A (MSRA) and B3 (MSRB3), iodothyronine deiodinase 1 (DIO1), 2 (DIO2), and 3 (DIO3), glutathione peroxidase 1 (GPX1) and 4 (GPX4), thioredoxin reductase 1 (TXNRD1) and 3 (TXNRD3), and metallothionein 3 (MT3) in the liver, as well as the potential impact on the FA profile of breast muscle of four- and six-week-old broilers fed diets with low and high levels of Se-yeast and Cd.

## 2. Materials and Methods

### 2.1. Animals, Diets, and Experimental Procedures

A total of 300, one-day-old, as-hatched Cobb broiler chickens from a commercial hatchery were used. Five replicate pens of four dietary treatments were employed, namely T1, T2, T3, and T4, randomly allocated in the house (the pen was the experimental unit). Each replicate was assigned to a clean concrete floor pen (2 m^2^), and chickens were raised on a litter of wheat straw shavings. There were 15 broilers per pen and 75 per treatment. In treatment T1, birds were fed a diet supplemented with 0.3 ppm Se (Se-yeast), without added Cd; in treatment T2, broilers were fed a diet with 0.3 ppm Se and 10 ppm Cd; in treatment T3, they were fed a diet with 0.3 ppm Se and 100 ppm Cd; in treatment T4, broilers were fed a diet with 3 ppm Se and 100 ppm Cd. The Cd was added to diets T2, T3, and T4 in the form of CdCl_2_ (Sigma-Aldrich, St Louis, MO, USA). Supplemented Se was obtained from a yeast source, Sel-Plex^®^ (Alltech Inc., Nicholasville, KY, USA).

The trial protocol was approved by the Institutional Committee for Animal Use and Ethics of the Faculty of Animal Science and Aquaculture of the Agricultural University of Athens (Ethical protocol code: 27-20032014). During the whole trial, chickens were handled in compliance with national and European Union (EU) regulations and laws and in accordance with the guidelines and principles for the care of animals in experimentation [23].

The duration of the experiment was 42 days and broilers were raised, according to Cobb’s management manual, in a house where ventilation and light were controlled. The lighting program was a 23-h/1-h of light/dark cycle, while heat was provided using a heating lamp in each pen. The birds were fed a starter diet up to the 10th day of their life, then a grower diet up to the 20th day, and a finisher diet up to the 42nd day (Table 1). Water and food were provided *ad libitum*. At the end of the 28th and 42nd days, two chickens per pen were killed in order to obtain tissue samples from breast muscle and liver, which were rapidly frozen in liquid nitrogen and kept at −80 °C until analyzed.

### 2.2. Determination of Fatty-Acid Profile

The feeds were prepared by grinding through a 1-mm screen, whereas breast samples were carefully freed from any visible adipose and connective tissues, and then blended in short bursts in a domestic food processor. Extraction and methylation of FA in feed and breast muscle samples were performed according to the method of O’Fallon et al. [24]. Then, 1-g (± 0.05) duplicate samples were hydrolyzed for 1.5 h at 55 °C in 6 mL of a mixture containing 5.3 mL of methanol and 0.7 mL of 10 N KOH. A known amount (ca. 0.5 mg) of internal standard (C13:0) was added to the samples prior to hydrolysis. Subsequently, 0.58 mL of 24 N H_2_SO_4_ was added to the samples, which were then incubated at 55 °C for 1.5 h to prepare the fatty-acid methylesters (FAME). Hexane (3 mL) was added to the reaction tube, which was vortex-mixed and centrifuged at 1100× *g*. The supernatant hexane layer containing the FA methyl esters was evaporated to dryness under nitrogen stream, rediluted in 0.5 mL of clear hexane, and kept at −20 °C until analyzed by gas chromatography.

A temperature-programmed run was followed on a Perkin Elmer Autosystem XL gas chromatograph equipped with a 30 m × 0.25 mm × 0.25 μm internal diameter HP-Innowax capillary column (Agilent Technologies, J&W GC columns, Santa Clara, CA, USA) and a flame ionization detector (FID; Perkin Elmer, Waltham, MA, USA), as described elsewhere [22]. Commercial standards (FAME 37 Component; Sigma-Aldrich Co. Supelco, St. Louis, IL, USA) were used to identify the FAs, which were quantified using the amount of internal standard added prior to hydrolysis. The sum of areas for all FA peaks compared to that for the internal standard was used to calculate the total weights of FAs (in mg/100 g) in diets and breast muscle. Individual FAs were expressed as percentage by weight of total FA.

### 2.3. RNA Isolation and Reverse Transcription

Total RNA was extracted from approximately 50 mg of tissue using TriReagent (Sigma-Aldrich Co., St. Louis, IL, USA) according to the manufacturer’s instructions. Extracted RNA was treated with DNAseI (New England Biolabs, Beverly, MA, USA) according to the manufacturer’s instructions. The quality and quantity of the RNA extracted were confirmed by spectrophotometry, as well as gel electrophoresis. Total RNA (500 ng) was reverse transcribed with the PrimeScript First-Strand complementary DNA (cDNA) Synthesis Kit (Takara Bio Inc, Kusatsu, Shiga 525-0058, Japan), according to the manufacturer’s instructions, using a mix of random hexamers and oligo-dT primers.

### 2.4. Real-Time Quantitative PCR

Relative levels of mRNA for target genes (*CAT*, *SOD1*, *SOD2*, *MSRA*, *MSRB3*, *DIO1*, *DIO2*, *DIO3*, *GPX1*, *GPX4*, *TXNRD1*, *TXNRD3,* and *MT3*) were quantified with real-time quantitative PCR using SYBR Green chemistry (Life Technologies, Austin, TX, USA). The amount of sample RNA was normalized using glyceraldehyde 3-phosphate dehydrogenase (*GAPDH*), β-actin (*ACTB*), and hypoxanthine phosphoribosyltransferase 1 (*HPRT1*) as housekeeping genes. A pair of primers for each of the genes used in this study was constructed using PERLprimer software (version 1.1.21) [25], except for β-actin, where a primer pair designed by Iqbal et al. [26] was used. Details for all primers used are found in Table 2. PCR reactions were performed in the 7500 Real Time system (Applied Biosystems, Foster City, CA, USA) using the SYBR^®^ Select Master Mix (Life Technologies, Austin, TX, USA) according to the manufacturer’s protocol. Each reaction (10 μL) contained 5 ng of RNA equivalent, as well as 300 nmol·L^−1^ forward and reverse primers for each gene. Reactions were performed in duplicate using the following thermal protocol: 2 min at 95 °C, and 40 cycles of 15 s at 95 °C and 60 s at 60 °C. A melt curve analysis was performed in order to confirm reaction specificity. Relative gene expression of selected targets was determined by applying a modified version of the Pfaffl [27] normalization method against the three housekeeping genes [28].

### 2.5. Statistical Analysis

Data for FA were analyzed using the SPSS statistical package (version 17.0, IBM Corp., Armonk, NY, USA.). A generalized linear model (GLM) for repeated measures with treatment and age as fixed effects and their interactions was carried out. Post hoc tests were performed for diet when appropriate, using Tukey’s multiple range test, and significance was set at 0.05. All data for FAs are presented as least-squares (LS) means.

Statistical analysis of gene expression was performed using Prism version 6.0 (GraphPad Software, Inc., La Jolla, CA, USA). Normal distribution of data values was assessed using the D’Agostino–Pearson omnibus test. For variables that did not exhibit a normal distribution, common logarithmic (*MSRB3, DIO3, GPX1, GPX4, TXNRD1*) or cube-root (*MSRA, CAT, SOD2, DIO2, MT3*) transformation was used in order to meet ANOVA assumptions. Data were analyzed using two-way analysis of variance (ANOVA). “Age” and “treatment” were the two independent variables, while further analysis of the interaction between the two independent variables was also carried out. Tukey’s honest significant difference (HSD) post hoc tests were performed to analyze the effect of “treatment” within “age”. Data are presented as means ± standard error for variables that did not require transformation to meet ANOVA assumptions. Data for transformed variables are presented as back-transformed means with back-transformed upper and lower confidence limits (confidence interval 95%). The level of statistical significance was set at *p* < 0.05.

## 3. Results

### 3.1. Breast Muscle Fatty-Acid Composition

Significant differences in the breast muscle FA composition between treatments were observed in the present study. Total weights of FA were lower (*p* < 0.05) in T3 and T4 when compared to T1 and T2 broiler chickens. Regarding the FA profile of breast meat, total *n*-6 FAs were lower (*p* < 0.05) in T3 and T4 broilers compared to T1 and T2 broiler chickens, mainly as a result of the decrease in 18:2*n*-6 (Table 3). Total *n*-3 FAs were not affected by the dietary treatment; however, changes in individual FA occurred. Most notably, 18:3*n*-3 decreased (*p* < 0.05) and 20:5*n*-3 increased (*p* < 0.05) in breast muscle of broilers from T3 and T4, respectively, compared to T1 and T2. Dietary treatments did not affect total saturated fatty acids (SFAs). In contrast, total PUFAs were strongly affected by the dietary treatments; T3 and T4 had significantly lower (*p* < 0.05) PUFAs and, as a result, a lower PUFA/SFA ratio (*p* < 0.05), in comparison with T1 and T2 broiler chickens (Table 3). Sampling time (age of broilers) had significant effects on monounsaturated fatty acids (MUFAs), PUFAs, and *n*-3 FAs. Total MUFAs (in particular *cis9*-18:1) were lower (*p* < 0.001), whereas PUFAs and *n*-3 FAs were greater (*p* < 0.005) at 42 days compared to 28 days of age (Table 3). No significant differences in the breast muscle FA composition between T1 and T2, or between T3 and T4 were found.

### 3.2. Gene Expression of Liver Antioxidant Enzymes

**Figure 1:**
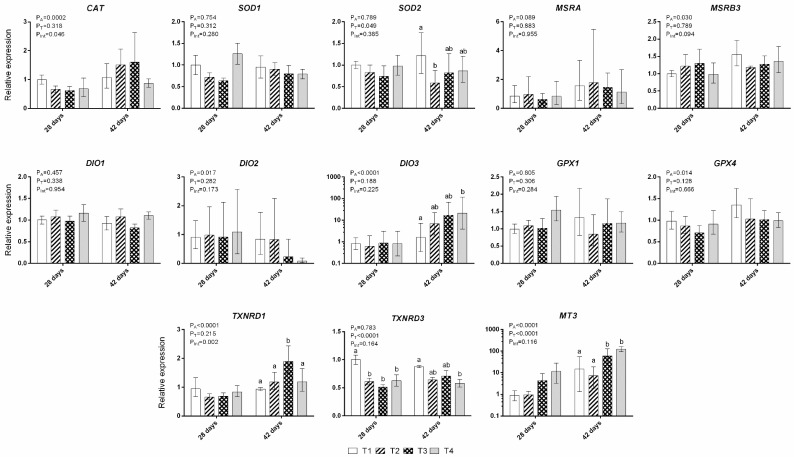
Relative expression levels of catalase (*CAT*), superoxide dismutase 1 (*SOD1*) and 2 (*SOD2*), methionine sulfoxide reductase A (*MSRA*) and B3 (*MSRB3*), iodothyronine deiodinase 1 (*DIO1*), 2 (*DIO2*), and 3 (*DIO3*), glutathione peroxidase 1 (*GPX1*) and 4 (*GPX4*), thioredoxin reductase 1 (*TXNRD1*) and 3 (*TXNRD3*), and metallothionein 3 (*MT3*). Data for *SOD1*, *DIO1*, and *TXNRD3* are presented as means ± standard error of the mean (SEM). Data for all other genes are presented as back-transformed means with back-transformed upper and lower confidence limits. Significances of main factor effects “age” (P_A_) and “treatment” (P_T_), as well as their interaction (P_Int_), are reported for each variable. Columns with different superscripts within the same age differ significantly (*p* < 0.05).

Overall, age of broilers (28 vs. 42 days) was a major factor significantly affecting gene expression of *CAT* (*p* < 0.001), *MSRB3* (*p* < 0.05), *DIO2* (*p* < 0.05), *DIO3* (*p* < 0.0001), *GPX4* (*p* < 0.05), *TXNRD1* (*p* < 0.0001), and *MT3* (*p* < 0.0001) (Figure 1). Similarly, treatment effects were found to be statistically significant for gene expression of *SOD2* (*p* < 0.05), *TXNRD3* (*p* < 0.0001), and *MT3* (*p* < 0.0001). Furthermore, post hoc multiple comparisons within the same age group revealed that the effect was pronounced on the 42-day-old broilers. In detail, there was a downregulation of *SOD2* expression in the T2 treatment when compared to the control T1 group, with no statistically significant differences for the other two treatments (T3 and T4). Regarding *TXNRD3*, T4 treatment samples exhibited lower levels of expression when compared to those of the control T1 group. As for *MT3* expression, it was highly upregulated (5–10-fold) in T3 and T4 treatments when compared to treatments T1 and T2. Additionally, *DIO3* expression in the liver increased incrementally (T1 < T2 < T3 < T4), although those differences were found to be statistically significant only between T4 and T1 treatments. Finally, another important finding was the observed interaction of the two main factors in the expression of *CAT* and *TXNRD1*. More specifically, *CAT* expression was upregulated in groups T2 and T3 in 42-day-old broilers when compared to 28-day-old broilers, but not in groups T1 and T4. The exact same effect was observed for *TXNRD1*. Regarding the expression of *SOD1*, *MSRA*, *DIO1*, and *GPX1*, no statistically significant effect was observed for any of the main factors or their interaction (Figure 1).

## 4. Discussion

The study was designed taking into account that (i) the EU limit of Cd in feed is 5 ppm [29], (ii) in some cases, Cd levels in feed exceed maximum permitted limits [30], (iii) increased essential element supplementation may alleviate the negative effects of toxic metal contamination [15,31,32], and (iv) the maximum permitted inclusion level of Se is 0.3 ppm (in the United States) [33]. The present study investigated the interactions between Cd, Se at low or high concentrations (well below toxic levels) [34], and gene expression of several enzymes that affect the antioxidant/prooxidant balance, finding that gene expression is altered in the presence of prooxidants and that organic Se can protect the body during stress conditions induced by Cd.

It is known that selenomethionine, a large constituent of Se-yeast, is non-specifically incorporated into proteins replacing methionine [35], and stored tissue Se amounts can be mobilized and employed in circumstances of oxidative stress. Cadmium is absorbed from the gastrointestinal tract and lungs and is mainly accumulated in the liver and kidney, where it is bound to metallothionein (MT) [36,37]. Metallothioneins are among the most well-known antioxidants that protect against metal toxicity [38,39]. They are low-molecular-weight proteins (~7 kDa) that bind to and are induced by free cytosolic metal ions, especially Cd, Zn, Cu, and Hg, and are implicated in the defense against metal toxicity [40]. These properties indicate that MT is an important factor affecting metal toxicity in vertebrates as it was previously shown in invertebrates [41]. Indeed, *MT3* expression was highly increased after exposure to 100 ppm Cd, and this increase persisted even after high Se addition. Moreover, *MT3* expression was significantly increased with age (28 vs. 42 days of experimental time), i.e., when Cd was accumulated in the liver [19]. Selenium was shown to lower the accumulation of toxic elements by shifting the distribution of tissue elements from MTs toward high-molecular-mass proteins [42,43]. Generally, it seems that Cd exposure markedly increases the expression of MTs in various species, with some differences depending on the Cd concentration and the duration of exposure [44]. Our findings collectively support the hypothesis that Cd increases the synthesis of MT, which plays a role in heavy-metal detoxification by reducing oxidative stress [45].

Thioredoxin reductase (TXNRD) enzymes are oxidoreductases that use NADPH to catalyze the reduction of oxidized thioredoxin (Trx) [46]. There are three mammalian TXNRDs: cytoplasmic/nuclear TXNRD1 which reduces Trx1, mitochondrial TXNRD2 which reduces Trx2, and testes-specific TXNRD3. Their functions are essential part of the Trx system, redox regulation, antioxidant defense, and cell signaling [12,47]. It was shown that increased Se intake results in increased expression of both the TXNRD1 and TXNRD2 proteins. However, the levels of Se are not the only regulator of expression of TXNRDs. Their expression may be affected, under certain conditions, by oxidative stress or as a response to growth factors [47]. In our study, TXNRD3 mRNA expression was significantly decreased after Cd addition, and this effect could not be reversed by Se.

Superoxide dismutase, as well as GPX, CAT, and metal-binding proteins (transferrin, metallothionein, albumin, myoglobin, haptoglobin, hemopexin, ceruloplasmin, ferritin, and lactoferrin), belongs to the first line of defense developed by the antioxidant system of the cell in order to prevent the initial formation of free radicals. Superoxide dismutase 2 inactivates catalysts or removes precursors of free radicals [14]. Addition of 10 ppm Cd caused a downregulation of *SOD2* expression in T2 treatment when compared to the control T1 group. It is possible that the absence of an effect of high Se addition between T3 and T4 on SOD2 gene expression was related with its use either for the formation of Se–Cd complexes [19,48] or for antioxidant protection against the induced oxidation by Cd [49]. Similar results were obtained by previous studies in chicken kidney [50] and liver [7], where significantly decreased SOD activities were demonstrated after 60 days of dietary co-treatment with Se and Cd.

Interestingly, the results of this study also pointed that, with experimental time and the accompanying increased levels of Cd in the liver [19], mRNA expression of CAT, DIO3, GPX4, and TXNRD1 was elevated, probably as a protective attempt of the antioxidant defense system against oxidative stress caused by Cd [14].

In relation to the above, Se contribution on metabolic pathways associated with the protection of the organism against oxidative stress was shown to induce changes in the activity of selenoproteins. Their expression is regulated, among others, by the concentration of this element [51]. However, Se concentration does not obligatorily affect the rate of transcription of selenoprotein genes. The observed differences in protein expression may be the result of changes in mRNA translation or reduced stability (increased degradation). Depending on Se levels, various effects were observed on cellular functions (energy metabolism, immunity) [52].

In this work, the protective mechanism of Se could not be sufficiently clarified by mRNA expression alone, since Cd itself could have induced the synthesis of antioxidant proteins [53]. Previously, it was shown that Cd was able to modify the activity of some enzymes, both with antioxidant and other activities, interacting directly with the enzymatic proteins [54]. In this case, the observed Se-induced expression differences at the mRNA level of the proteins in question could have no effect on the toxic action of Cd. The results from these studies, however, are not easily comparable with the results of the present study, since they refer to different examined proteins, different animal species (rats vs. chickens), different routes of administration (intraperitoneal (i.p.) injection vs. dietary addition), and different levels of administration, among others.

Administration of selected trace elements, such as Se, as a remedy to ameliorate Cd toxicity, was lately evaluated in several studies [55,56,57]. Selenium exhibited protective effects against Cd toxicity by counteracting the immunosuppressive, as well as hepatic and renal oxidative, damage [58]. Previous works showed that supranutritional levels of supplemented Se were able to prevent the peroxidation of PUFAs in broiler chicken meat, thereby increasing their intramuscular levels [22]. It is known that Cd present in feeds results in oxidative deterioration of lipids [4] and, therefore, it is not unlikely that it can reduce the PUFAs in meat. In the present study, meat PUFA composition decreased in broiler chickens fed with a 100 mg Cd/kg diet; however, it cannot be attributed to Cd-induced oxidation alone. Broiler chickens fed with 100 mg Cd/kg diet had significantly lower feed intake, as found in our previous work [1], which resulted in a lower energy, as well as PUFA intake and deposition in the breast muscle. The lower energy intake was clearly demonstrated by the lower body weight [1] and the reduction in breast muscle fatness, as indicated by the total weights of FA herein, which may explain the lower PUFA composition in broiler chickens fed with 100 mg Cd/kg. However, muscle fatness plays an important role in defining the FA profile [59], and the differences in the breast muscle FA composition between treatments were evaluated by removing the effects of fatness using the total weights of FA as a covariate in the statistical analysis. Breast meat PUFAs, and in particular *n*-6 PUFAs, remained lower in broiler chickens fed 100 ppm Cd, when compared to those fed the diet unsupplemented with Cd (T1) or that supplemented with 10 ppm Cd. These results indicate that an oxidative stress imposed by dietary Cd addition at very high levels cannot be dismissed. Hence, it appears that the reduction in PUFA composition may be caused by both the lower feed and PUFA intake and the increased oxidation processes induced by the presence of Cd at very high dietary levels. Furthermore, a protective role of dietary Se on FA composition was observed. The addition of 10 ppm Cd to the diet (T2 diet containing 0.3 ppm Se) did not alter the FA profile of breast meat in comparison with the diet unsupplemented with Cd (T1). This indicated that the dietary Se addition at the recommended levels (0.3 ppm) in T2 was adequate to alleviate any potential negative effects of low amounts of Cd. However, dietary Se addition either at the recommended (0.3 ppm) or at supranutritional levels (3.0 ppm) could not ameliorate the impact of high Cd levels. These results are in agreement with our previous findings, which showed that broilers fed supplemental Se can tolerate low levels of Cd present in the diet; however, they cannot tolerate high levels of Cd in the diet regardless of the level of dietary Se. Most notably, it was shown that dietary 10 ppm Cd addition (T2) did not result in any significant change in chicken body mass compared with that of chickens fed diets without added Cd (T1), while addition of 100 ppm Cd significantly reduced (*p* < 0.001) chicken body mass compared with that of chickens fed no added Cd [1]. The body mass of chickens fed a diet with 100 ppm Cd and 3 ppm Se (T4) did not differ compared with that of chickens fed 100 ppm Cd and 0.3 ppm Se, and it was lower compared with that of chickens fed no or low levels of added Cd.

## 5. Conclusions

It seems that a direct implication of the oxidative stress status of the liver on the regulation of MT3, SOD2, and TXNRD3 gene expression, along with FA profile changes, in breast muscle exists; however, the exact mechanisms of these interactions need to be further investigated. Based on previous reports from our group and on the present findings on the effect of Se supply on the oxidative stress balance in chickens exposed to Cd, our data would suggest that dietary Se can ameliorate the negative effects of Cd, but cannot be efficient in the presence of excessive dietary amounts of Cd.

## Figures and Tables

**Table 1:** Composition (g∙kg^−1^) and calculated analysis of the experimental broiler diets.

Ingredients (g∙kg^−1^)	Starter (0–10 days)	Grower (11–20 days)	Finisher (21–42 days)
Maize	559.5	631.0	658.4
Soybean meal	333.7	261.0	226.4
Gluten	50.0	50.0	50.0
Soybean oil	15.4	16.9	25.7
Dicalcium phosphate	15.1	14.4	13.2
Limestone	16.1	15.7	14.8
Lysine	0.2	1.5	2.1
Methionine	1.1	1.3	1.5
NaCl	4.9	4.2	3.9
Premix1	4.0	4.0	4.0
**Calculated Analysis**			
Metabolizable energy (ME) (MJ∙kg^−1^)	12.5	12.9	13.3
Crude protein (CP) (g∙kg^−1^)	210.0	190.0	180.0
Sodium (g∙kg^−1^)	2.0	1.7	1.6
Ca (g∙kg^−1^)	10.0	9.6	9.0
Available phosphorus (g∙kg^−1^)	5.0	4.8	4.5
Methionine (g∙kg^−1^)	5.3	5.1	5.2
Methionine + cystine (g∙kg^−1^)	8.9	8.4	8.2
Lysine (g∙kg^−1^)	12.0	11.0	10.5
**Fatty-Acid (FA) Profile (% of total FA)**			
14:0	0.08	0.08	0.09
16:0	13.27	13.28	13.35
*cis7*-16:1	0.04	0.05	0.04
*cis9*-16:1	0.15	0.15	0.10
17:0	0.10	0.09	0.10
17:1	0.05	0.05	0.05
18:0	3.15	3.18	3.39
*cis9*-18:1	26.51	27.20	26.64
18:2*n*-6	48.70	48.39	47.90
18:3*n*-3	4.06	3.66	4.14
20:0	0.42	0.40	0.44
20:1*n*-9	0.33	0.30	0.30
20:2*n*-6	0.08	0.07	0.09

**Table 2:** Sequences and relative positions of forward (F) and reverse (R) primers for catalase (CAT), superoxide dismutase 1 (SOD1) and 2 (SOD2), methionine sulfoxide reductase A (MSRA) and B3 (MSRB3), iodothyronine deiodinase 1 (DIO1), 2 (DIO2), and 3 (DIO3), glutathione peroxidase 1 (GPX1) and 4 (GPX4), thioredoxin reductase 1 (TXNRD1) and 3 (TXNRD3), metallothionein 3 (MT3), glyceraldehyde 3-phosphate dehydrogenase (GAPDH), β-actin (ACTB), and hypoxanthine phosphoribosyltransferase 1 (HPRT1) used in real-time qPCR. NCBI—National Center for Biotechnology Information; ID—identifier; F—forward; R—reverse.

Gene Name (*Gallus gallus* NCBI Transcript ID)	Primer Name	Sequence 5′ to 3′	Position in Transcript
*CAT* (NM_001031215.2)	F-CAT gal	TCCACGTTAAGACCGATCAG	839–858
	R-CAT gal	AATGTAGAAAGACCAGGAAGGA	960–981
*SOD1* (NM_205064.1)	F-CuZnSOD gal	ATGCAGATAGGCACGTGG	229–246
	R-CuZnSOD gal	ACTGCCATCTTAAGCATTTCAG	474–495
*SOD2* (NM_204211.1)	F-MnSOD gal	AGCCTAAAGGAGAATTGATGGA	365–386
	R-MnSOD gal	CAGCAATGGAATGAGACCTG	551–570
*MSRA* (XM_004935891.1)	F-MSRA gal	CAAAGAAGTCTGTTCAGGTTTAGG	359–382
	R-MSRA gal	GCATTCCTTGTGTCGGGT	559–576
*MSRB3* (NM_001199578.1)	F-MSRB3 gal	GACGGAAAGTGCCTTTGAG	253–271
	R-MSRB3 gal	GGACTGATCACATCATAGAAGGA	377–399
*DIO1* (NM_001097614.1)	F-DIO1 gal	GCTCACGCAGTAGATGGA	404–421
	R-DIO1 gal	CACTCCTCCCTTGTAGATGAC	605–625
*DIO2* (NM_204114.3)	F-DIO2 gal	TACAAGCAGGTCAAACTTGGA	806–826
	R-DIO2 gal	CACACTTGCCACCAACAC	897–914
*DIO3* (NM_001122648.1)	F-DIO3(2) gal	CATGTTGATTTGCCAGCCA	942–960
	R-DIO3(2) gal	AACAGATCCCGAAGGAAGAG	1078–1097
*GPX1* (NM_001277853.1)	F-GPX1 gal	AACCAATTCGGGCACCAG	254–271
	R-GPX1 gal	CCAGATGATGTACTGCGGG	457–475
*GPX4* (AF498316.2)	F-GPX4 gal	TGTGGAAGTGGCTGAAGG	378–395
	R-GPX4 gal	CTCGATCACGTAAGGATCCTC	500–520
*TXNRD1* (NM_001030762.2)	F-TXNRD1 gal	AGGAATACAATACTGTGCTGCT	1014–1035
	R-TXNRD1 gal	TGAACTAACAGTCTTCCTGCC	1213–1233
*TXNRD3* (NM_001122777.1)	F-TXNRD3 gal	TGAGAAGAATGGGAAAGTACCTG	1344–1366
	R-TXNRD3 gal	CGTTGATATAGTCACACTTTGTGG	1499–1522
*MT3* (NM_001097538.1)	F-MT3 gal	ACGTGTGGAGACAACTGC	42–59
	R-MT3 gal	GCACACTTGGCACATCCT	119–136
*GAPDH* (NM_204305.1)	F-GAPDH gal	GCTGAATGGGAAGCTTACTG	720–739
	R-GAPDH gal	AAGGTGGAGGAATGGCTG	918–935
*ACTB* (NM_205518.1)	F-ACTB gal	TGCTGTGTTCCCATCTATCG	152–171
	R-ACTB gal	TTGGTGACAATACCGTGTTCA	281–301
*HPRT1* (NM_204848.1)	F-HPRT1 gal	CGGCCAGACTTTGTTGGA	569–586
	R-HPRT1 gal	ACCAGAGTTGAAGCCTGTG	768–786

**Table 3:** Effects of treatment and age on the total weights of fatty acids (mg/100g wet tissue) and the fatty-acid (FA) profile (% of total FAs) of breast meat (adjusted for total weights of FAs).

	Treatment (T) ^1^				RMSE ^2^	Day of Age (D)		RMSE ^2^	*p*-Value		
	T1	T2	T3	T4		28	42		T	D	T × D
Total weights of FA	996.33 ^a^	910.14 ^a^	609.93 ^b^	597.72 ^b^	92.882	799.38	757.68	65.677	<0.001	0.528	0.171
14:0	0.38	0.39	0.45	0.44	0.025	0.43	0.40	0.018	0.067	0.038	0.002
14:1	0.09 ^a^	0.10 ^ab^	0.12 ^b^	0.11 ^ab^	0.009	0.11	0.09	0.006	0.067	0.001	0.111
15:0	0.08	0.07	0.06	0.07	0.006	0.07	0.07	0.004	0.191	0.822	0.342
16:0	20.55	20.26	21.15	19.96	0.521	20.51	20.45	0.367	0.135	0.865	0.622
*cis*7-16:1	0.37 ^a^	0.37 ^a^	0.40 ^ab^	0.46 ^b^	0.031	0.47	0.33	0.022	0.022	<0.001	0.041
*cis*9-16:1	2.98 ^a^	3.35 ^ab^	4.04 ^b^	3.89 ^b^	0.298	4.15	2.98	0.210	0.012	<0.001	0.312
17:0	0.10	0.09	0.09	0.09	0.006	0.08	0.11	0.004	0.452	<0.001	0.581
17:1	0.20 ^ab^	0.19 ^a^	0.25 ^b^	0.24 ^ab^	0.018	0.22	0.23	0.013	0.011	0.278	0.692
18:0	9.28	8.65	9.05	9.14	0.276	8.68	9.38	0.194	0.129	0.001	0.663
*cis*9-18:1	23.00	22.96	23.26	24.71	0.948	24.77	22.19	0.668	0.273	<0.001	0.468
*cis*11-18:1	3.21 ^a^	3.22 ^a^	3.47 ^ab^	3.71 ^a^	0.160	3.63	3.18	0.113	0.025	<0.001	0.673
18:2*n*-6	18.64 ^a^	18.95 ^a^	16.49 ^b^	16.05 ^b^	0.620	17.34	17.73	0.437	<0.001	0.375	0.585
18:3*n*-6	0.11	0.14	0.13	0.13	0.011	0.13	0.13	0.007	0.152	0.730	0.776
18:3*n*-3	0.74 ^ab^	0.79 ^a^	0.63 ^b^	0.73 ^ab^	0.039	0.78	0.67	0.028	0.004	<0.001	0.013
20:0	0.05	0.05	0.15	0.07	0.071	0.05	0.11	0.050	0.477	0.312	0.506
20:1*n*-9	0.49	0.45	0.41	0.47	0.043	0.55	0.36	0.030	0.337	<0.001	0.634
20:2*n*-6	0.82 ^a^	0.85 ^a^	0.54 ^b^	0.50 ^b^	0.045	0.72	0.64	0.032	<0.001	0.010	0.077
20:3*n*-6	1.30	1.35	1.46	1.46	0.074	1.42	1.37	0.052	0.178	0.353	0.118
20:4*n*-6	4.30	4.35	3.69	3.67	0.403	3.30	4.70	0.284	0.299	<0.001	0.967
20:5*n*-3	0.29 ^a^	0.31 ^ab^	0.40 ^bc^	0.44 ^c^	0.031	0.33	0.39	0.022	<0.001	0.015	0.325
22:2*n*-6	1.84	1.75	1.66	1.65	0.109	1.80	1.66	0.077	0.438	0.074	0.908
22:4*n*-6	1.09 ^a^	1.13 ^a^	0.73 ^b^	0.71 ^b^	0.069	0.78	1.05	0.049	<0.001	<0.001	0.256
22:5*n*-3	0.64	0.64	0.58	0.63	0.058	0.50	0.74	0.041	0.714	<0.001	0.807
22:6*n*-3	0.51	0.53	0.52	0.59	0.047	0.50	0.57	0.033	0.451	0.035	0.723
SFA^3^	30.50	29.56	31.00	29.86	0.570	29.89	30.57	0.402	0.063	0.093	0.853
MUFA^3^	30.29	30.58	31.86	33.54	1.294	33.81	29.32	0.912	0.119	<0.001	0.391
PUFA^3^	30.28 ^a^	30.77 ^a^	26.83 ^b^	26.55 ^b^	1.020	27.59	29.63	0.719	<0.001	0.006	0.823
PUFA:SFA	1.00 ^ac^	1.04 ^a^	0.87 ^b^	0.89 ^bc^	0.042	0.93	0.97	0.030	0.001	0.141	0.697
*n*-3^4^	2.19	2.26	2.14	2.38	0.115	2.11	2.37	0.081	0.183	0.002	0.606
*n*-6^4^	22.98 ^a^	23.31 ^a^	20.47 ^b^	20.00 ^b^	0.639	21.45	21.92	0.450	<0.001	0.300	0.533

Means with different superscripts differ significantly (*p* < 0.05; Tukey’s test). ^1^ T1 = 0.3 ppm Se/0 ppm Cd, T2 = 0.3 ppm Se/10 ppm Cd, T3 = 0.3 ppm Se/100 ppm Cd, T4 = 3 ppm Se/100 ppm Cd; ^2^ RMSE = root-mean-square error; ^3^ SFA = total saturated fatty acids (12:0 + 14:0 + 15:0 + 16:0 + 17:0 + 18:0 + 20:0), MUFA = total monounsaturated fatty acids (14:1 + *cis*7-16:1 + *cis*9-16:1 + 17:1 + *cis*9-18:1 + *cis*11-18:1 + 20:1*n*-9 + 22:1*n*-9), PUFA = total polyunsaturated fatty acids (18:2*n*-6 + 18:3*n*-3 + 18:3*n*-6 + 20:2*n*-6 + 20:3*n*-6 + 20:4*n*-6 + 20:5*n*-3 + 22:2*n*-6 + 22:4*n*-6 + 22:5*n*-3 + 22:6*n*-3); ^4^ *n*-3 = 18:3*n*-3 + 20:5*n*-3 + 22:5*n*-3 + 22:6*n*-3, *n*-6 = 18:2*n*-6 + 18:3*n*-6 + 20:2*n*-6 + 20:3*n*-6 + 20:4*n*-6 + 22:2*n*-6 + 22:4*n*-6.

## References

[1] Al-Waeli A., Zoidis E., Pappas A., Demiris N., Zervas G., Fegeros K. (2013). The role of organic selenium in cadmium toxicity: Effects on broiler performance and health status. Animal.

[2] Xu F., Liu S., Li S. (2015). Effects of selenium and cadmium on changes in the gene expression of immune cytokines in chicken splenic lymphocytes. Biol. Trace Elem. Res..

[3] Hussain T., Shukla G.S., Chandra S.V. (1987). Effects of cadmium on superoxide dismutase and lipid peroxidation in liver and kidney of growing rats: In vivo and in vitro studies. Pharmacol. Toxicol..

[4] Shaikh Z.A., Vu T.T., Zaman K. (1999). Oxidative stress as a mechanism of chronic cadmium-induced hepatotoxicity and renal toxicity and protection by antioxidants. Toxicol. Appl. Pharmacol..

[5] Waisberg M., Joseph P., Hale B., Beyersmann D. (2003). Molecular and cellular mechanisms of cadmium carcinogenesis. Toxicology.

[6] Zhao W., Liu W., Chen X., Zhu Y., Zhang Z., Yao H., Xu S. (2014). Four endoplasmic reticulum resident selenoproteins may be related to the protection of selenium against cadmium toxicity in chicken lymphocytes. Biol. Trace Elem. Res..

[7] Li J.L., Jiang C.Y., Li S., Xu S.W. (2013). Cadmium induced hepatotoxicity in chickens (*Gallus domesticus*) and ameliorative effect by selenium. Ecotoxicol. Environ. Saf..

[8] Li J.L., Gao R., Li S., Wang J.T., Tang Z.X., Xu S.W. (2010). Testicular toxicity induced by dietary cadmium in cocks and ameliorative effect by selenium. Biometals.

[9] Rahman M.M., Hossain K.F.B., Banik S., Sikder M.T., Akter M., Bondad S.E.C., Rahaman M.S., Hosokawa T., Saito T., Kurasaki M. (2019). Selenium and zinc protections against metal-(loids)-induced toxicity and disease manifestations: A review. Ecotoxicol. Environ. Saf..

[10] Pappas A.C., Zoidis E., Surai P.F., Zervas G. (2008). Selenoproteins and maternal nutrition. Comp. Biochem. Physiol..

[11] Zoidis E., Pappas A.C., Georgiou C.A., Komaitis E., Fegeros K. (2010). Selenium affects the expression of GPx4 and catalase in the liver of chicken. Comp. Biochem. Physiol..

[12] Zoidis E., Seremelis I., Kontopoulos N., Danezis G. (2018). Selenium-dependent antioxidant enzymes: Actions and properties of selenoproteins. Antioxidants.

[13] Messaoudi I., Banni M., Said L., Said K., Kerkeni A. (2010). Involvement of selenoprotein P and GPx4 gene expressions in cadmium-induced testicular pathophysiology in rat. Chem. Biol. Interact..

[14] Pappas A.C., Zoidis E., Fegeros K., Zervas G., Surai P.F., Pappas A.C., Zoidis E., Fegeros K., Zervas G., Surai P.F. (2010). Cadmium Toxicity and the Antioxidant System. Environmental Health-Physical, Chemical and Biological Factors Series.

[15] Jihen E.H., Imed M., Fatima H., Abdelhamid K. (2008). Protective effects of selenium (Se) and zinc (Zn) on cadmium (Cd) toxicity in the liver and kidney of the rat: histology and Cd accumulation. Food Chem. Toxicol..

[16] Lazarus M., Orct T., Jurasoviae J., Blanuša M. (2009). The effect of dietary selenium supplementation on cadmium absorption and retention in suckling rats. Biometals.

[17] Mahan D.C., Lyons T.P., Jacques K.A. (1999). Organic selenium: Using nature’s model to redefine selenium supplementation for animals. Biotechnology in the Feed Industry.

[18] Mahmoud K.Z., Edens F.W. (2003). Influence of selenium sources on age related and mild heat stress-related changes of blood and liver glutathione redox cycle in broiler chickens (*Gallus domesticus*). Comp. Biochem. Physiol..

[19] Al-Waeli A., Pappas A.C., Zoidis E., Georgiou C.A., Zervas G., Fegeros K. (2012). The role of selenium in cadmium toxicity: Interactions with essential and toxic elements. Br. Poult. Sci..

[20] Chen X., Zhu Y.H., Cheng X.Y., Zhang Z.W., Xu S.W. (2012). The protection of selenium against cadmium-induced cytotoxicity via the heat shock protein pathway in chicken splenic lymphocytes. Molecules.

[21] Liu L.L., Li C.M., Zhang Z.W., Yao H.D., Xu S.W. (2014). Protective effects of selenium on cadmium-induced brain damage in chickens. Biol. Trace Elem. Res..

[22] Pappas A.C., Zoidis E., Papadomichelakis G., Fegeros K. (2012). Supranutritional selenium level affects fatty acid composition and oxidative stability of chicken breast muscle tissue. J. Anim. Physiol. Anim. Nutr..

[23] NRC (National Research Council) (1996). Guide for the Care and Use of Laboratory Animals.

[24] O’Fallon J.V., Busboom J.R., Nelson M.L., Gaskins C.T. (2007). A direct method for fatty acid methyl ester synthesis: Application to wet meat tissues, oils, and feedstuffs. J. Anim. Sci..

[25] Marshall O.J. (2004). PerlPrimer: Cross-platform, graphical primer design for standard, bisulphite and real-time PCR. Bioinformatics.

[26] Iqbal M., Philbin V.J., Withanage G.S.K., Wigley P., Beal R.K., Goodchild M.J., Barrow P., McConnell I., Maskell D.J., Young J. (2005). Identification and functional characterization of chicken Toll-like receptor 5 reveals a fundamental role in the biology of infection with Salmonella enterica serovar Typhimurium. Infect. Immun..

[27] Pfaffl M.W. (2001). A new mathematical model for relative quantification in real-time RT-PCR. Nucleic Acids Res..

[28] Hellemans J., Mortier G., De Paepe A., Speleman F., Vandesompele J. (2007). qBase relative quantification framework and software for management and automated analysis of real-time quantitative PCR data. Genome Biol..

[29] EC 2005 (2002). Commission Directive 05/87/EC Amending Annex I to Directive 2002/32/EC of the European Parliament and of the Council on undesirable substances in animal feed as regards lead, fluorine and cadmium. Off. J. Eur. Union.

[30] Li Y.X., Xiong X., Lin C.Y., Zhang F.S., Li W., Han W. (2010). Cadmium in animal production and its potential hazard on Beijing and Fuxin farmlands. J. Hazard. Mater..

[31] Nolan T.D., Brown D. (2000). The influence of elevated dietary zinc, selenium, and their combination on the suppressive effect of dietary and intraperitoneal cadmium on egg production in laying hens. J. Toxicol. Environ. Health.

[32] Jemai H., Messaoudi I., Chaouch A., Kerkeni A. (2007). Protective effect of zinc supplementation on blood antioxidant defense system in rats exposed to cadmium. J. Trace Elem. Med. Biol..

[33] (2018). Food and Drug Administration. CFR–Code of Federal Regulations Title 21. https://www.accessdata.fda.gov/scripts/cdrh/cfdocs/cfcfr/cfrsearch.cfm?fr=573.920.

[34] Gad M.A., Abd El-Twab S.M. (2009). Selenium toxicosis assessment (in vivo and in vitro) and the protective role of vitamin B12 in male quail (Coturnix coturnix). Environ. Toxicol. Pharmacol..

[35] Rayman M.P. (2004). The use of high-selenium yeast to raise selenium status: how does it measure up?. Br. J. Nutr..

[36] Banni M., Messaoudi I., Saïd L., El Heni J., Kerkeni A., Saïd K. (2010). Metallothionein gene expression in liver of rats exposed to cadmium and supplemented with zinc and selenium. Arch. Environ. Contam. Toxicol..

[37] Klaassen C.D., Liu J., Diwan B.A. (2009). Metallothionein protection of cadmium toxicity. Toxicol. Appl. Pharmacol..

[38] Chan H.M., Cherian M.G. (1992). Protective roles of metallothionein and glutathione in hepatotoxicity of cadmium. Toxicology.

[39] Klaassen C.D., Liu J. (1998). Metallothionein transgenic and knock-out mouse models in the study of cadmium toxicity. J. Toxicol. Sci..

[40] Park J.D., Liu Y., Klaassen C.D. (2001). Protective effect of metallothionein against the toxicity of cadmium and other metals. Toxicology.

[41] Xie L., Klerks P.L. (2004). Metallothionein-like protein in the least killifish Heterandria Formosa and its role in cadmium resistance. Environ. Toxicol. Chem..

[42] Underwood E.J., Suttle N.F., Underwood E.J., Suttle N.F. (1999). The Mineral Nutrition of Livestock.

[43] Wangher P.D. (2001). Selenium and the brain: a review. Nutr. Neurosci..

[44] Choi H.J., Ji J., Chung K.H., Ahn I.Y. (2007). Cadmium bioaccumulation and detoxification in the gill and digestive gland of the Antarctic bivalve Laternula elliptica. Comp. Biochem. Physiol..

[45] Baudrimont M., Andres S., Durrieu G., Boudou A. (2003). The key role of metallothioneins in the bivalve Corbicula fluminea during the depuration phase, after in situ exposure to Cd and Zn. Aquat. Toxicol..

[46] Holmgren A. (1989). Thioredoxin and glutaredoxin systems. J. Biol. Chem..

[47] Liang Y., Lin S.L., Wang C.W., Yao H.D., Zhang Z.W., Xu S.W. (2014). Effect of selenium on selenoprotein expression in the adipose tissue of chickens. Biol. Trace Elem. Res..

[48] El-Sharaky A.S., Newairy A.A., Badreldeen M.M., Eweda S.M., Sheweita S.A. (2007). Protective role of selenium against renal toxicity induced by cadmium in rats. Toxicology.

[49] Surai P.F. (2006). Selenium in Nutrition and Health.

[50] Liu L., Yang B., Cheng Y., Lin H. (2015). Ameliorative effects of selenium on cadmium-induced oxidative stress and endoplasmic reticulum stress in the chicken kidney. Biol. Trace Elem. Res..

[51] Lammi M., Qu C. (2018). Selenium-Related transcriptional regulation of gene expression. Int. J. Mol. Sci..

[52] Fernandes J., Hu X., Smith M.R., Go Y.M., Jones D.P. (2018). Selenium at the redox interface of the genome, metabolome and exposome. Free Radic. Biol. Med..

[53] Casalino E., Calzaretti G., Landriscina M., Sblano C., Fabiano A., Landriscina C. (2007). The Nrf2 transcription factor contributes to the induction of alpha-class GST isoenzymes in liver of acute cadmium or manganese intoxicated rats: Comparison with the toxic effect on NAD(P)H:quinone reductase. Toxicology.

[54] Casalino E., Calzaretti G., Sblano C., Landriscina C. (2000). Cadmium-dependent enzyme activity alteration is not imputable to lipid peroxidation. Archiv. Biochem. Biophys..

[55] McCarty M.F. (2012). Zinc and multi-mineral supplementation should mitigate the pathogenic impact of cadmium exposure. Med. Hypotheses.

[56] Zhang R., Wang Y., Wang C., Zhao P., Liu H., Li J., Bao J. (2017). Ameliorative effects of dietary selenium against cadmium toxicity is related to changes in trace elements in chicken kidneys. Biol. Trace Elem. Res..

[57] Branca J.J.V., Morucci G., Maresca M., Tenci B., Cascella R., Paternostro F., Ghelardini C., Gulisano M., Di Cesare Mannelli L., Pacini A. (2018). Selenium and zinc: Two key players against cadmium-induced neuronal toxicity. Toxicol. In Vitro.

[58] El-Boshy M.E., Risha E.F., Abdelhamid F.M., Mubarak M.S., Hadda T.B. (2015). Protective effects of selenium against cadmium induced hematological disturbances, immunosuppressive, oxidative stress and hepatorenal damage in rats. J. Trace Elem. Med. Biol..

[59] Wood J.D., Enser M., Fisher A.V., Nute G.R., Sheard P.R., Richardson R.I., Hughes S.I., Whittington F.M. (2008). Fat deposition, fatty acid composition and meat quality: A review. Meat Sci..

